# Mining protein function from text using term-based support vector machines

**DOI:** 10.1186/1471-2105-6-S1-S22

**Published:** 2005-05-24

**Authors:** Simon B Rice, Goran Nenadic, Benjamin J Stapley

**Affiliations:** 1 Faculty of Life Sciences, University of Manchester, UK; 2 School of Informatics, University of Manchester, UK; 3 National Centre for Text Mining, Manchester, UK

## Abstract

**Background:**

Text mining has spurred huge interest in the domain of biology. The goal of the BioCreAtIvE exercise was to evaluate the performance of current text mining systems. We participated in Task 2, which addressed assigning Gene Ontology terms to human proteins and selecting relevant evidence from full-text documents. We approached it as a modified form of the document classification task. We used a supervised machine-learning approach (based on support vector machines) to assign protein function and select passages that support the assignments. As classification features, we used a protein's co-occurring terms that were automatically extracted from documents.

**Results:**

The results evaluated by curators were modest, and quite variable for different problems: in many cases we have relatively good assignment of GO terms to proteins, but the selected supporting text was typically non-relevant (precision spanning from 3% to 50%). The method appears to work best when a substantial set of relevant documents is obtained, while it works poorly on single documents and/or short passages. The initial results suggest that our approach can also mine annotations from text even when an explicit statement relating a protein to a GO term is absent.

**Conclusion:**

A machine learning approach to mining protein function predictions from text can yield good performance only if sufficient training data is available, and significant amount of supporting data is used for prediction. The most promising results are for combined document retrieval and GO term assignment, which calls for the integration of methods developed in BioCreAtIvE Task 1 and Task 2.

## Background

Rapid advancements in biology, molecular biology and biomedicine have led to the development of a range of factual and experimental databases. Apart from structured data related to sequences, expressions, etc., the significant body of biomedical knowledge is stored in the domain literature. The size of textual archives is increasing so rapidly that it is impossible for any user to locate and assimilate new knowledge without automated help. In particular, efficient curation of biological databases relies essentially on the ability to search and manage published articles rapidly and cost-effectively. This fact has spurred huge interest in designing text mining methods that can help users in locating, collecting and extracting relevant knowledge represented in an unstructured, free-text format.

The BioCreAtIvE evaluation was organised to assess the performance of current text mining systems in two tasks. Task 1 was protein name identification in free text (for different species, namely fly, mouse and yeast) [1,2]. Task 2 addressed a set of related tasks based on functional annotation of human proteins by assigning relevant Gene Ontology (GO) terms from a corpus of full-text documents [3]. Task 2 was generally designed to be analogous to the process by which expert annotators curate and update records in resources such as Swiss-Prot and genomic databases. It was divided in three subtasks. Task 2.1 addressed the problem of selecting relevant textual evidence from a given document to support the annotation of a given protein name with a given GO term (i.e. "find a statement in the text that motivates this database annotation"), assuming that the document is relevant for both the protein and the GO term in question. Task 2.2 was about selecting both a relevant GO term and a corresponding text segment from a given document for a given protein ("assign a GO term and support it by a statement from text"). As in Task 2.1, the relevance of the document was also presupposed in Task 2.2. Finally, Task 2.3 represented the most complete, realistic and challenging problem amongst the three sub-tasks. In Task 2.3, systems were expected to assign appropriate GO terms and to select relevant passages for a given protein identifier, but relevant documents had to be identified automatically from a given corpus.

**Figure 1:**
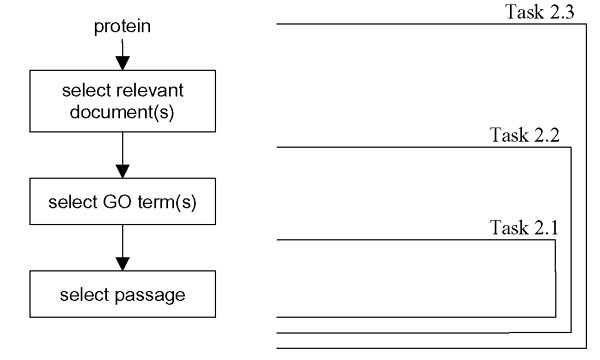
**Subtasks in BioCreAtIvE Task 2. **We considered Task 2 subtasks as nested problems: for solving Task 2.3, tasks 2.2 and 2.1 are approached as subtasks.

In this article we present our approach used within the framework of BioCreAtIvE Task 2. We participated in all three subtasks. We considered Task 2.3 as the most general one, and the other two subtasks as its nested problems (see Figure 1): for solving Task 2.3, tasks 2.2 and 2.1 were approached as subtasks. More precisely, to solve Task 2.2, we need only a method to assign relevant GO terms, and once we have obtained them, we can apply the methods from Task 2.1 to retrieve supporting text. Similarly, for Task 2.3 we need to solve the document retrieval problem, and once we have relevant documents selected, the methods from Task 2.2 can be employed.

Although the main idea of BioCreAtIvE Task 2 was to provide help for human annotators with the time-consuming curation of biological databases (i.e. to provide textual passages that are "sufficient" for reading in order to make or confirm an annotation), we approached the task as a knowledge discovery problem. More precisely, our approach aims at mining associations from a corpus even when an explicit statement (and thus a textual evidence) relating a protein to a GO term is not present (in a given set of documents). The approach follows an original and renowned example of "hidden links" presented by Swanson and Smalheiser [4-6] (see also more recent work on various statistical and machine-learning methods, using co-occurrence frequency counts, different similarity and correlation measures, document themes, etc. [7-12]). We have previously developed related methods for combined retrieval and classification of a protein's sub-cellular location using support vector machines (SVMs) and a bag-of-words approach [13], as well as for classification of yeast genes using eleven classes of the upper part of the GO ontology [14]. The approach we used for BioCreAtIvE Task 2 was largely derived from that work.

In the remainder of the paper we firstly describe the methods that were used to solve the task. Then, we present and discuss the evaluation and results.

## Methods

We employed a supervised machine learning approach to assignment of GO terms to proteins, together with an extensive terminological processing of documents (which aimed at generation of relevant features for classification and protein annotation). We based our method on SVMs, which have been demonstrated to perform well at the document classification task [15], as we construed the protein function assignment task as a modified form of this problem (cf. also [13,14]). The approach is mainly based on the idea that biological entities (represented by domain terms) that co-occur in text with a protein of interest are indicative of its function, and that proteins with similar co-occurrences of terms have related roles. Consequently, learning relevant and informative co-occurring terms for a given GO term should give clues for assignment of that GO term to proteins that have similar distributional patterns.

Assignments of GO terms (both for learning and predicting) were based on collecting "weak" co-occurrence evidence within documents, rather than on explicit statement(s) of protein function. Therefore, an important facet of our approach was that GO assignments were not derived from a single, "relevant" passage or sentence, but from document(s) relevant to a given protein. Further, selection of supporting passages (as minimal retrieval units we used paragraphs as tagged in an SGML-tagged version of distributed documents) was based on a similar idea. Each paragraph pertaining to a given protein was assessed with respect to a given GO term, and the highest scoring passage was selected. More specifically, the employed method involved three steps: a) pre-processing of documents and feature selection, b) training the SVMs on the released training data, and c) predicting GO terms and selection of paragraphs for target (testing) genes.

### a) Document pre-processing, feature selection and weighting

For Task 2, a corpus of around 30,000 full-text SGML-encoded documents (containing around 70 million words) from the Journal of Biological Chemistry was distributed by the organisers. Document pre-processing involved several steps. Firstly, we parsed the SGML documents to remove *experimental*, *methods *and *reference *sections of each document *body*, as we considered that these were unlikely to contain information on protein function and might introduce unnecessary noise. We also removed non-textual elements (such as tables and figures), but retained figure legends, as they might have some useful information and clarification (cf. also [16]). All remaining SGML tags (apart from paragraph tags) were removed (tags for *abstracts *were treated as paragraph tags). The problem with *sup *tags (used for both superscripts (e.g. in names) and for marking references to footnotes) was ignored, i.e. these tags were removed and their content concatenated to the preceding text (which generated obvious problems in case of footnotes). SGML entities (such as encodings of Greek letters, e.g. *&alpha;*) were replaced with the appropriate ASCII equivalents (e.g. *alpha*), collected from available DTDs. Then, the whole corpus was POS tagged using a general-language tagger (EngCG [17] was used).

Extraction of features used for protein classification (i.e. association to GO terms) was the next step. As features, we used automatically extracted terms (or terminologically relevant sequences) that co-occurred with a given protein within the same document. Since terms represent the most important concepts in a domain, we hypothesised that they might be useful features for the annotation task. Also, we have previously shown that using biological terms as classification features improves performance when compared to single words [14]. However, terms from controlled vocabularies (e.g. from the GO ontology) are extremely sparse in free text (cf. [14,18,19]), not only due to lexical variation but mainly because they are typically descriptors rather than real names i.e. terms (e.g. *ligase activity, forming phosphoric ester bonds*, GO:0016886). Further, the high neology rate of other relevant terms makes existing glossaries incomplete for dynamic searching, and thus automatic term extraction tools are needed for efficient term identification [20].

In order to automatically recognise terms in text, we used an enhanced version of the C-value method [21]. The method has been previously used to recognise terminology in many biomedical sub-domains (e.g. in the domain of nuclear receptors [22] or from yeast corpora [14]). The input to the original C-value is a POS tagged corpus, and the output is a list of suggested terms, ranked according to their likelihood of representing relevant domain concepts. We modified the method so that the information on paragraphs and documents in which recognised terms appeared was also produced.

In order to suggest possible terms, the C-value method combines linguistic formation patterns and statistical analysis. The linguistic part includes extraction of term candidates by using a set of formation patterns, and a stop list to eliminate frequent false term candidates. Term formation patterns act as linguistic filters to a POS tagged corpus: filtered sequences are considered as potential realisations of domain concepts. For the BioCreAtIvE task we used the simplest term formation pattern (namely, noun phrases following the pattern (A|N)^+ ^N, where A and N denote an adjective and a noun respectively), which has proven to have the best precision/recall ratio [21,22] for the biomedical domain. Note that this approach can also collect terms from controlled vocabularies (e.g. from the GO ontology) that appeared in text, but we did not give any extra credits to such terms (see also discussion).

**Figure 2:**
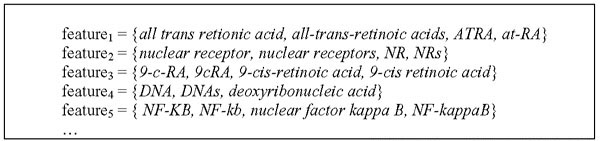
**Examples of terminological features (synterms). **Various lexical representations of terms were conflated in sets called synterms, which were used as features for assignment of protein function.

**Figure 3:**
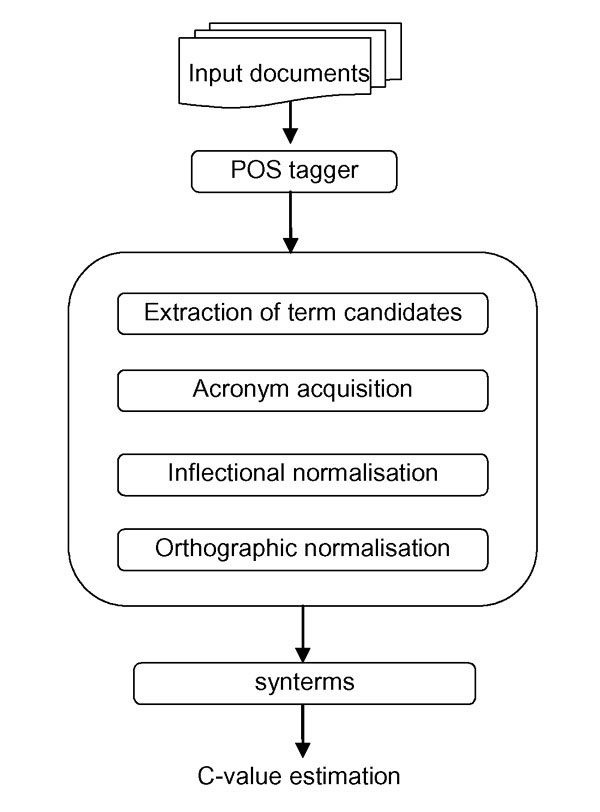
**Extracting terminological features. **Term features were extracted through several steps, including extraction of term candidates and acronyms, their inflectional and orthographic normalisation, and estimation of termhoods for synterms.

Since many biological concepts are designated by more than one surface lexical representation, extracted term candidates were post-processed and normalised in order to link equivalent term variants (and thus possibly neutralise variations of various term occurrences). In order to conflate equivalent surface expressions, we carried out linguistic normalisation of individual term candidates (see [23,24] for details). For the BioCreAtIvE task, we normalised typo-orthographic (*leukaemia *and *leukemia*; *amino-acid *and *amino acid*) and inflectional variants (*nuclear receptor *and *nuclear receptors*; *Down's syndrome *and *Down syndrome*). Each term candidate was then mapped to a normalised canonical representative (CR), and consequently we established an equivalence relation, where two term candidates were related if and only if they shared the same CR. The partitions of this relation are denoted as *synterms *(synonymous terms): a synterm contains various surface term representations sharing the same CR. Further, an acronym recognition and conflation module [23] was used to link acronyms and their variants to respective CRs (e.g. both *NFKB factor *and *NF-KB *are linked to *nuclear factor kappa B*), and these were also included in corresponding synterms (see Figure 2 and Figure 3 for an illustration of the overall process of extracting terminological features).

Finally, synterms were statistically analysed, and each set was assigned likelihood to represent a domain specific concept (for details see [24]). Termhood (called C-value) of a given synterm is based on a statistical measure that amalgamates the cumulative frequency of occurrence of its elements in the corpus, the frequency of occurrence of its CR as a form nested within other CRs (of other synterms), the number of such candidate synterms, and the average length of term variants included in the synterm. Synterms that were top-ranked (first 75%) according to their C-values were selected as features for classification. Using synterms (which conflate term variants) as features instead of individual term representations aims at neutralisation of lexical variation across documents and authors (i.e. it is equivalent if a protein co-occurs with either *NF-KB *or *NFKB factor *or with *nuclear factor kappa B *– the same synterm will be used/assigned as a feature in each case). For Task 2, we extracted 1.4 million distinct synterms from the corpus, and stored them in a database (along with their global and local document/paragraph frequencies needed for weighting the features).

For weighting synterm features, we used a form of inverse document frequency (*idf*, [25,26]) that took account of the number of documents considered relevant to a given protein. More precisely, the weight of a feature *w *for protein *p *is given by



where *R*_*p *_is a set of relevant documents for the protein *p*, *f*_*j*_*(w) *is the frequency of *w *in document *j*, and *N*_*w *_is the global frequency of *w *in the whole collection of documents. Note that the assignment of relevant documents was based on the released training data for the learning phase, while for the prediction phase we used either pre-selected documents (tasks 2.1 and 2.2) or we employed a retrieval method (Task 2.3).

### b) Learning a SVM for each GO term

In the learning phase, we trained SVMs for GO terms on term vectors formed from document-protein pairs from the initially released training data. For Task 2, the training data included 1858 triples of the form (*GO term*, *protein*, *document*), meaning that assignment of the *GO term *to the *protein *is supported by the *document*, with 659 distinct GO terms (assigned to 755 proteins and supported by 638 documents). This means that we had on average only 2.82 examples per GO term. Therefore, for each GO term, we collected positive learning examples from all training triples with GO terms that matched or were descendant from the GO term concerned. For these examples, we collected relevant documents (as released in the training set) and corresponding term-features (found in these documents) were used as pertaining to the given GO term. The negative training examples were obtained by taking an equivalent number of examples (and assigned documents) from sibling GO terms and their children.

Apart from the released GO terms, we trained additional GO classifiers for a whole subtree of GO formed from the root to GO terms occurring in the training data (for each node in the subtree, we collected positive examples released for all of its descendants). Using the hierarchy of the GO, we were able to extend the original 659 GO classifiers to create the total of 1436 (which still covers less than 10% of nodes of the GO ontology).

The SVM classifiers were trained using the *svm-light *package [27], and support vectors (for each GO term) were stored in a database for efficient access in the next phase. More precisely, as the decision function in SVM classification is the weighted sum of the kernel function evaluated between a test case and support vectors, this means that we can evaluate the decision function within the database management system. Once trained, the classification system was entirely contained within the database which greatly simplified calculation of predictions (in particular when one has to manage a large number of classifiers).

Note, however, that – while we had the training data for assignments of GO terms to proteins – there were no data provided for training on selection of relevant paragraphs (only whole documents were distributed). Therefore, we used the same SVMs for both problems (prediction of GO terms for proteins and selection of passages).

### c) Prediction of GO terms and selection of passages

The prediction of GO terms for a target protein was performed in two steps. First we created a feature vector for a given target protein by collecting all synterms from given/relevant document(s). For tasks 2.1 and 2.2, the set of relevant documents for a given protein was pre-selected (i.e. specified as part of the task), while for Task 2.3 we employed an ad-hoc retrieval method to obtain documents from the corpus (see below). In the second step, we tested the feature vector against respective GO classifier(s), and selected the GO term(s) associated with the top-ranked classifiers (i.e. the classifiers whose decision function values were the highest).

A similar approach was employed for detection of relevant passages: each paragraph pertaining to a given protein (i.e. appearing in any of the protein's relevant documents) was formed into a term vector and tested against the relevant GO classifier(s). The highest scoring passage was assigned as pertaining to the protein/GO annotation. Note that here we did not consider "similarity" between paragraphs and a testing protein, i.e. the relevant paragraph was selected only as being related to the GO term in question (but the documents from which the paragraphs were taken were assumed to be relevant to the protein).

More specifically, the testing procedures for each of the subtasks were as follows:

#### Task 2.1: selection of supporting passages from a specified document for a given GO term

For a given (*protein*, *GO term*) pair we tested all the paragraphs from the specified *document *against the corresponding GO classifier (i.e. classifier that corresponds to the given *GO term*), and the top-ranked paragraph was selected. However, note that we could select supporting paragraphs only for the GO terms that appeared in training examples or were on a path from a training GO term to the root. We generated two submissions for this subtask. In Submission 1, if we did not have the corresponding GO classifier trained, the testing example was skipped. In Submission 2, we used a classifier trained for a nearest neighbour GO term if the exact one was not available, in an attempt to improve recall. Informally, we climbed up through the GO hierarchy from the given GO term until we found an available classifier. More formally, the nearest neighbour node was selected as the lowest common ancestor (in the same branch) for the test *GO term *and the original training data, and it was used to select a relevant passage.

#### Task 2.2: prediction of GO terms and selection of supporting passages from a specified document

For a given protein, we generated its feature vector from a specified, pre-selected document, and tested it against all available GO classifiers. We then selected GO terms corresponding to the top-ranked classifiers (the number of assigned GO terms was as required by the assessors). Then, for each (*protein*, *GO term*) pair obtained, we applied the procedures used in Task 2.1, Submission 1, in order to select a relevant paragraph (this was Task 2.2, Submission 1). We also generated Submission 2, where we used (additional) GO classifiers derived from a new training set composed jointly from the initially released training data for Task 2 and the test data for Task 2.1 (580 GO terms, assigned to 138 proteins). We reasoned that additional classifiers might improve recall, and since the test sets for tasks 2.1 and 2.2 were distinct, we believed this was a fair approach to obtain more training data. We re-trained the SVM classifiers (as explained above) with the new data, obtaining additional 582 GO classifiers (2018 in total, including those obtained by the propagation through the GO hierarchy). So, in Submission 2 we used the same methodology as in Submission 1, but with additional GO classifiers.

#### Task 2.3: prediction of GO terms and selection of supporting passages from a corpus

In this subtask, the main challenge was to retrieve a set of relevant documents for a testing protein. During the pre-processing phase, we collected human protein names from Swiss-Prot/Trembl, and stored them in a database (note that – for the methods that we employed – protein names were not needed for tasks 2.1 and 2.2). We implemented an ad-hoc retrieval approach that used a variant of the inverse document frequency weighting [25,26]. We scored each document in the corpus against a query formed from all the words of the DE (description) and GN (gene name) fields of the Swiss-Prot/Trembl entries of each testing protein. In addition, if a document contained an exact phrasal match to a (multi-word) term from the DE field, the weight contribution from this term was raised to a power proportional to the length of this match. Once top-ranked documents were assigned to a given protein as its relevant documents, the methods from Task 2.2 were applied to assign relevant GO terms and retrieve supporting passages. Analogously to Task 2.2, we generated two submissions; Submission 1 with the original training GO classifiers, and Submission 2 with the data from Task 2.1 used as additional training examples.

## Evaluation and results

Evaluation of the results was performed by database curators from the European Bioinformatics Institute (EBI). It is obvious that it was a huge challenge to define and apply a consistent and meaningful evaluation approach. In the accepted framework, the focus was mainly on "usefulness" of a selected passage for deriving a given GO annotation of a given protein. The passages were assessed both from the perspective of the relevance to the suggested GO term and the relevance to the protein in question. "*High*" judgments were assigned when GO terms or proteins were highly related to the selected passage, and "*low*" judgements were assigned when there was no relevance. In "*perfect*" predictions, both the GO term and the protein were marked as "high". If a passage was generally related to a given GO term (e.g. it was relevant for a more general or neighbouring GO term), the evaluators assessed such results as "*general*". In case of proteins, "*general*" marks were assigned to cases where the selected passage was not exactly relevant for a specific protein, but was relevant to the protein family or a homologue. We present the results for each subtask separately.

### Task 2.1: selection of supporting passages from a specified document for a given GO term

There were 1076 test examples in this subtask. The results were modest (see Table 1). In general, only one quarter of selected paragraphs were deemed highly relevant for either a GO term or for a protein, or for both. Note that, however, there was a high discrepancy i.e. low overlap between the testing and training sets of GO terms: 43% of the testing examples for this subtask referred to a GO term that did not occur in the training set (even when terms from the suggested propagation through the GO hierarchy were included), and even 50% of the distinct GO terms in the testing data were absent from the available training data. Since we used a supervised machine learning approach, we were clearly unable to make judgements related to testing examples containing such GO terms. Still, by using a classifier of a more general, nearest-neighbour GO term when no classifier for the actual GO term was available (Submission 2), we substantially improved recall (the number of relevant paragraphs more than doubled, from 106 to 232) without sacrificing precision (see Table 1). Compared to submissions from other participants, precision of our Submission 2 predictions was poor (for "perfect" predictions we were ranked 16^th ^out of 21 submissions), but recall was ranked in the upper half (9^th ^out of 21 submissions). For predictions marked as "general", precision of all participating systems was in the range 5–6%, with recall for our system in the top 6 (out of 21).

### Task 2.2: prediction of GO terms and selection of supporting passages from a specified document

There were 435 test examples (i.e. (*protein*, *document*) pairs) in this subtask. The results for this subtask were disappointing. In Submission 1 (only the initial training data was used) precision was only 2.6% (see Table 2). The inclusion of classifiers derived from Task 2.1 data used as additional training examples (Submission 2), improved both precision and recall substantially (more than three times each), which indicates that the method might be more effective if the coverage of training data was broader. Still, we believe that one of the main causes of such poor performance was the lack of data on which the predictions were based (GO term assignments in this case were based only on a single specified document). Compared to submissions from other participants, in this subtask we were ranked as 6^th ^and 12^th ^(out of 18 submissions) for "general" and "perfect" predictions respectively, while our system was 4^th ^and 9^th ^with respect to recall (for "general" and "perfect" respectively).

### Task 2.3: prediction of GO terms and selection of supporting passages from a corpus

Only 10 testing proteins have been distributed for this task, and – in our case – only 5 of them (with total of 36 and 52 predictions for two submissions) were evaluated. Therefore, the assessment of performance in Task 2.3 and comparison to subtasks 2.1 and 2.2 are limited. Still, the results for Task 2.3 were encouraging: on average, 50% of assigned GO terms and selected passages were deemed relevant for a given protein (see Table 3, Submission 1). In Submission 2, we used additional training data (from Task 2.1) in order to improve recall. However, we only got more predictions, with recall remaining almost the same, which consequently decreased precision. For this subtask we were unable to compare our performance to other systems, as such results were not available.

In this subtask, the prediction of GO terms was based on evidence that has been collected from several documents (the average number of documents retrieved for each protein was 10), and not from a single article as in subtasks 2.1 and 2.2. Still, the results of Task 2.3 were quite variable for individual proteins. For two proteins (Q9972 and P08247), precision of assigned GO terms was very high (more than 70% of predictions were highly relevant, see Table 4), while it was very poor for other two proteins (P30153 and Q9BYW1; also see Table 4). We believe that a plausible reason for such discrepancy was the relevance of retrieved documents: for the first two proteins, almost all collected documents were relevant to the proteins (and thus we had a substantial body of relevant information to make predictions), while for the second pair, only few retrieved documents were related to the proteins in question (making it difficult to capture relevant information). We further discuss this below.

## Discussion

To our mind, there were at least two separate problems that were part of Task 2: assignment of GO terms that describe protein function (tasks 2.2 and 2.3), and selection of a supporting passage for a given (*protein*, *GO term*) pair (all subtasks, in particular Task 2.1). Also, an additional, non-trivial problem is automatic retrieval of relevant documents (Task 2.3). The results obtained in response to these problems are quite different: in many cases we have relatively good assignment of GO terms to proteins (in particular when several relevant documents have been retrieved for a given protein), but the selected paragraphs are typically non-relevant.

### Prediction of GO terms

For protein function assignments, we rely on capturing a substantial body of relatively weak evidence from document(s), rather than on a rare but explicit statement of protein function. Therefore, it is essential to obtain substantial data to support predictions (see also below). For example, Task 2.3 results show that – when several relevant documents are used to make predictions – significantly better performance can be achieved (e.g. compared to the Task 2.2 results). The average number of documents retrieved for each protein in Task 2.3 was 10, compared to a single document approach in Task 2.2. Thus, the results from Task 2.3 show that relevant latent information can be inferred from weak evidence when several documents are analysed, and that single documents are not always sufficient to automatically predict specific associations using the method we applied: assignments that have been derived from many documents were more reliable than assignments based on a single document. Further, while supporting documents improve precision of GO annotations, availability of training data can improve the overall performance (for example, the evaluation shows that relying on additional training examples in solving Task 2.2 (Submission 2) resulted in significantly improved precision and recall of predicted GO terms).

### Selection of supporting text

Selection of a relevant passage to support a (*protein*, *GO term*) pair is a huge challenge, as – in particular for assignment of functional annotations – supporting information can be distributed over several paragraphs or documents. From a biologist's point of view, short passages often cannot give unambiguous assignment of function without domain knowledge gleaned from other sources. Existing resources, for example Swiss-Prot, typically provide whole documents as supporting evidence. Analogously, the BioCreAtIvE training data also indicated only relevant documents. Therefore, as training examples for supporting passages were not available, it was impossible to automatically learn characteristic features on the paragraph level, and, consequently, we used SVM-classifiers trained on the document level. For the method that we applied, an additional problem (for predictions) was that passages (i.e. paragraphs) were typically too short, and consequently they contained few features (note that for proteins we used whole documents, containing more features). Therefore, as methods that rely on collecting sufficient amount of weak information cannot capture evidence from short textual segments, we could not provide accurate selection of paragraphs. Also, as longer passages have more features, our approach typically suggests lengthy paragraphs, rather than short sentences. Still, a promising outcome (as indicated by Task 2.1, Submission 2 results) is that using more general GO classifiers for passage extraction does not decrease precision.

### Retrieval of relevant documents

As indicated earlier, it is essential to provide an accurate set of relevant documents on which predictions will be based. To our mind, Task 2.3 (including document retrieval) is the most interesting and realistic problem, as it does not require pre-selection of documents and GO terms for annotation of a certain protein. While GO terms could be assigned to proteins using some non-textual data-mining methods (e.g. homology searching), "pre-selection" of (a single) relevant document(s) that is "guaranteed" to be relevant to both the protein of interest and the GO term to be assigned is a highly challenging task. If such documents are selected automatically, analysis (or discovery) of relevant GO terms has to be taken into account as part of the process (which then converges towards Task 2.3), while manual pre-selection of such documents by human annotators (in order to be used in an automated system) is non-realistic. We further believe that it is rarely the case that a single document can be guaranteed to be self-contained and relevant to both the protein of interest and the GO term to be assigned. When human annotators derive functional annotations from such documents, they would almost certainly make use of significant background knowledge. This knowledge needs to be "captured" by an automated system in one way or another. This can only happen if there is a sufficient number of relevant documents to be considered for prediction, or if some additional knowledge source is used.

Our experiments have confirmed that selection of relevant (or "suitable") documents for a given protein is not a trivial task (it generally corresponds to BioCreAtIvE Task 1B [2]), but a task of great relevance for mining protein function. In order to retrieve relevant documents for Task 2.3, we firstly experimented with exact dictionary look-up (using all synonyms from available databases) to match protein names in text. For 138 proteins from the list supplied in Task 2.1, we retrieved documents (from the BioCreAtIvE corpus containing 30,000 documents) for only 81 proteins (59%) using 446 available synonyms for these proteins from Swiss-Prot/Trembl. The main reason for such poor recall was extensive variability of protein names. Therefore, we implemented a retrieval method that did not rely exclusively on exact match, but also took into account individual words that comprised terms. The method worked well for simpler protein names (such as *synaptophysin *(P08247) and *BRCA1-associated RING domain protein 1 *or *BARD1 *(Q9972)), where relevant documents were retrieved with very high precision (above 90%, checked manually by in-house biologists). However, retrieved sets for complex protein names were typically not accurate, as documents were rarely related to proteins in question. For example, retrieval precision was around 10% for Task 2.3 proteins P30153 (*serine/threonine protein phosphatase 2A, 65 KDA regulatory subunit A, alpha isoform*) and Q9BYW1 (*solute carrier family 2, facilitated glucose transporter, member 11*). We believe that the relevance of the retrieval sets significantly influenced the quality of predictions for the respective proteins (see Table 4). An additional challenge for the retrieval of relevant documents is to ensure that the documents correspond to correct species. For example, in Task 2.3 in four cases (11%) we got high quality predictions, but the associated documents (and thus suggested paragraphs) were not about human proteins.

### Discovering and linking knowledge

Results of Task 2.3 have also shown that – when a sufficient body of documents is available – we were able to mine annotations from texts even when statements of certain relationships have not been clearly or explicitly stated. In such cases, relationships among proteins and GO terms were typically "discovered" by using a transitive closure of co-occurrence features collected from many documents (similarly to Swanson's approach [5]). For example, our method correctly linked the GO term *DNA-directed RNA polymerase II, holoenzyme *to the *BARD1 *protein (Q9972), although the documents in the protein's retrieval set did not contain an explicit statement of this relationship. Instead, the GO term was loosely linked to the *BRCA1 *protein in this set of documents, and using the co-occurrence of the two proteins (*BRCA1 *and *BARD1*) with other terms, we were able to mine the association between *BARD1 *and the GO term. The mined annotation was afterwards confirmed in an article with the explicit statement of the relationship [28], but this article was not present in the training and testing document collections used in BioCreAtIvE. Consequently, our approach suggested the annotation without the need that this relationship has been explicitly established and published in an article. Of course, it is obvious that in this case no appropriate supporting passages could be selected, as such information is distributed over several documents and is not presented explicitly.

This example illustrates that predictions (or hypothesis, in general) can be indeed mined and inferred from existing "hidden" and weak evidence that is present in literature, and not only from explicit statements. We believe that similar types of latent information are very common. For example, a statement of interaction between two proteins implies their cellular co-location; hence, knowing the location of one is sufficient evidence for location of the other. Further, some background knowledge can be used to infer additional associations. For example, evidence that a protein is involved in the *tricarboxylic acid cycle *implies that the protein is located in the mitochondria.

### Possible improvements

There is obviously significant room for improving the methods that we used for Task 2. For example, we treated all terms extracted from text equally, but additional credits could have been given to particularly relevant features for the task in question (i.e. GO terms in case of Task 2) when found as co-occurring with proteins/paragraphs of interest. Also, as we approached the protein function assignment task as a modified form of the document classification problem, the role of a protein was "limited" only to pre-selection of documents that would be analysed (for training a classifier for a given GO term, all documents that contained proteins annotated to the GO term were selected; for the annotation of a given protein, we collected only documents that contained mentions of the protein). Once the documents have been selected, training and classification were performed without further protein "input", and we used a whole document as a context surrounding a protein in question, from which the features (both for training and prediction) came. As documents may be too wide as relevant contexts, we will experiment with classification features that are collected from a narrower context of a protein (e.g. inside a paragraph where the protein is mentioned), or when feature weights depend on the distance from the protein occurrences. Still, spotting a mention of a protein in a paragraph is to a great extent a problem addressed in BioCreAtIvE Task 1, and thus integration of successful methods from that task could be beneficial. Also, this could help in locating relevant passages from the perspective of relevance to proteins (in the current implementation we did not measure the relevance of paragraphs to proteins in question). Furthermore, we believe that combination of our approach and some type of assessment of "lexical" similarity (e.g. one presented in [29]) between a paragraph (even a sentence) and a GO term entry can further improve the selection of relevant passages.

Additional knowledge and data sources might also be useful in improving the methods that we used for Task 2. In the current implementation, we approached the task in a "closed" manner, i.e. we relied *only *on released training resources, and no other data was used (apart from the GO hierarchy to generate classifiers for more general GO terms, and the Swiss-Prot database to obtain a list of protein names for Task 2.3). For example, we could have used a set of GO classifiers that we have previously trained on yeast data [14], and incorporated them into the generated pool of human classifiers, thus probably improving the coverage, or generated additional (non-perfect, but still high quality) training data using annotations from existing resources (similarly to a method suggested in [30]). Also, we could have used additional documents (not only from the released BioCreAtIvE corpus but also e.g. from Medline or from the Internet) to collect more features for released positive examples, thus possibly improving precision. Furthermore, additional documents could be used to support predictions (e.g. in Task 2.2). For example, we could have used a larger set of documents to make prediction of GO terms (instead of a single article – e.g. by retrieving documents relevant to the given protein from the whole corpus), but supporting paragraphs could be still selected from documents specified by the assessors. These possibilities are directions for our further experiments with the BioCreAtIvE data.

## Conclusion

Automatic extraction of concise information on protein function from literature is undoubtedly a task of great relevance and utility to molecular biologists. In our approach to mining protein function from text, we used SVMs and features derived from terms co-occurring with a given protein to assign GO terms. We approached the problem as a variant of document classification, where GO assignments were not derived from relevant passages, but from relevant documents.

The evaluation of the results shows the capabilities and limitations of supervised machine-learning approaches in text mining. Firstly, they can yield good performance only if sufficient training data is obtained, and significant amount of supporting data is used for prediction. The results show that performance improves as the number of relevant documents to a particular protein increases, while the method works poorly on short passages and/or single documents. The main reason is that short textual units often do not contain necessary information to infer protein function without information from other sources. This implies that our GO assignments (based on several documents) may be largely accurate and with relatively good recall, but finding the relevant passage may be difficult. Apart from a significant body of training examples (which needs to include examples of relevant text passages as well – if they are to be selected), such methods need to incorporate either some background knowledge (e.g. in the form of an ontology or a semantic network), or to analyse a substantial quantity of relevant text to learn or acquire such knowledge, or both. In our case, since we do not use any additional domain knowledge to support predictions, our method requires several documents for each protein in order to be effective. Of course, retrieval of relevant documents is an additional challenge, mainly because of lexical variability and ambiguity of protein names. On the other hand, we have shown that this approach can "discover" some associations from text even when an explicit statement relating a protein to a GO term is absent. In that sense, we believe that machine-learning approaches are more suited for addressing knowledge discovery tasks.

There is obviously space for future improvements and experiments as discussed in the previous Section. Despite moderate results, the BioCreAtIvE exercise was very valuable, in particular for the identification and clarification of user requirements and open challenges, and concrete progress on how best to evaluate and interpret the results of text mining. Further, distribution of correct testing data (in particular relevant supporting passages) will be one of the valuable results of the evaluation. Finally, one of the main lessons learnt is that the two BioCreAtIvE task should not be viewed as isolated problems: for a successful solution to BioCreAtIvE Task 2, successful methods from Task 1 will be of great help.

## Figures and Tables

**Table 1:** Task 2.1: precision of passage selection. The number and precision of selected passages (paragraphs) that were relevant to a given (*protein*,*GO term*) pair.

relevance to		Submission 1 submitted: 524 evaluated: 458		Submission 2 submitted: 998 evaluated: 896	
*GO term*	*protein*	passages	Precision	Passages	precision

high	high	59	12.9%	125	14.0%
high	general	19	4.1%	38	4.2%
general	high	28	6.1%	69	7.7%
**Total**		**106**	**23.1%**	**232**	**25.9%**

**Table 2:** Task 2.2: precision of GO term prediction and passage selection from a single specified document. The number and precision of selected pairs (*GO term*,*passage*) that were relevant to a given *protein*. The prediction of the GO terms was based only on a specified document.

relevance of		Submission 1 submitted: 502 evaluated: 502		Submission 2 submitted: 485 evaluated: 485	
*GO term*	*protein*	pairs	precision	Pairs	precision

high	high	3	0.6%	16	3.3%
high	general	2	0.4%	2	0.4%
general	high	8	1.6%	26	5.4%
**Total**		**13**	**2.6%**	**44**	**9.1%**

**Table 3:** Task 2.3: precision of GO term prediction and passage selection from a corpus. The number and precision of selected pairs (*GO term*,*passage*) that were relevant to a given *protein*. The prediction of GO terms was based on a set of retrieved documents.

relevance of		Submission 1 submitted: 54 evaluated: 36		Submission 2 submitted: 69 evaluated: 52	
*GO term*	*protein*	pairs	precision	pairs	precision

high	high	11	30.6%	11	21.2%
high	general	0	0%	0	0%
general	high	7	19.4%	6	11.5%
**Total**		**18**	**50.0%**	**17**	**32.7%**

**Table 4:** Results for specific proteins from Task 2.3. Individual prediction results for proteins evaluated in Task 2.3.

protein		predictions			
PAC	name	evaluated	high high	high general	general high

Q99728	*BRCA1-associated RING domain protein 1*	14	10 (71.4%)	0	4 (28.6%)
P08247	1.1.1.1.1 synaptophysin	3	0	0	3 (100%)
P30153	*serine/threonine protein phosphatase 2A, 65 KDA regulatory subunit A, alpha isoform*	6	1 (16.7%)	0	0
Q9BYW1	*solute carrier family 2, facilitated glucose transporter, member 11*	11	0	0	0
